# Monosodium Glutamate Intake, Dietary Patterns and Asthma in Chinese Adults

**DOI:** 10.1371/journal.pone.0051567

**Published:** 2012-12-11

**Authors:** Zumin Shi, Baojun Yuan, Gary A. Wittert, Xiaoqun Pan, Yue Dai, Robert Adams, Anne W. Taylor

**Affiliations:** 1 Department of Nutrition and Foodborne Disease Prevention, Jiangsu Provincial Centre for Disease Control and Prevention, Nanjing, China; 2 Discipline of Medicine, University of Adelaide, Adelaide, Australia; University of Ottawa, Canada

## Abstract

**Objectives:**

Emerging evidence shows that diet is related to asthma. The aim of this analysis was to investigate the association between monosodium glutamate (MSG) intake, overall dietary patterns and asthma.

**Methods:**

Data from 1486 Chinese men and women who participated in the Jiangsu Nutrition Study (JIN) were analyzed. In this study, MSG intake and dietary patterns were quantitatively assessed in 2002. Information on asthma history was collected during followed-up in 2007.

**Results:**

Of the sample, 1.4% reported ever having asthma. MSG intake was not positively associated with asthma. There was a significant positive association between ‘traditional’ (high loadings on rice, wheat flour, and vegetable) food pattern and asthma. No association between ’macho’ (rich in meat and alcohol), ‘sweet tooth’ (high loadings on cake, milk, and yoghurt) ‘vegetable rich’ (high loadings on whole grain, fruit, and vegetable) food patterns and asthma was found. Smoking and overweight were not associated with asthma in the sample.

**Conclusion:**

While a ‘Traditional’ food pattern was positively associated with asthma among Chinese adults, there was no significant association between MSG intake and asthma.

## Introduction

In many western countries (e.g. Australia, USA, and Canada), asthma is highly prevalent and affects more than 10% of adults [1]. Low socio-economic status (SES), female sex, smoking, and overweight are all associated with an increased prevalence of asthma [2]. In developing countries, the burden of asthma is less than in developed countries. In China, the prevalence of asthma was reported to be around 2% among adults [1], [3], [4]. Several hypotheses have been proposed to explain the greater burden of asthma in developed as compared to developing countries, including less indoor allergens exposure, improved hygiene and antibiotic usage (“hygiene hypothesis”) [5], obesity prevalence, and poor diet quality (low intake of dietary antioxidants) [6]. Occupational exposure can also lead to the development of asthma in adults [7]. A recent review indicates that nutrition may play a role in the etiology of asthma (e.g. dietary antioxidants, vitamin D, and maternal dietary intake during pregnancy) [8]. While some studies suggest a link between overall dietary pattern and asthma [9], [10], others do not [11], [12]. No study has reported on the association between dietary pattern and asthma in the Chinese population.

Monosodium glutamate (MSG) is a widely used food additive (flavour enhancer) especially in Asia [13]. In many Asian countries, MSG is added to dishes in a way similar to salt being used during home cooking. It is also widely used in many processed food in developed countries. In 1981, MSG was first reported by Allen and Baker to be linked to asthma attacks after ingesting a meal in a Chinese restaurant [14]. Small clinical trials on MSG and asthma have yielded inconsistent results [15]: some suggested that MSG intake was a trigger factor for asthma [16], [17], while others found no such association [18]–[20]. Studies with positive findings have been criticised for their research methods including lack of blinding of observers, inadequate baseline and control data, cessation of anti-inflammatory and bronchodilator medications before the challenge sequence [19]. After excluding nine non-randomized studies, based on two cross-over studies involving 24 adults, the latest Cochrane review concluded that there is no evidence to support the avoidance of MSG in adults with chronic asthma, yet data were limited and further research is needed [21]. In a recent study using an ovalbumin-sensitized asthma mouse model, Yoneda *et al* found that there were no adverse effects of continual ingestion of MSG on the development of asthma [22].

Currently there is no large population study assessing the relationship between MSG intake and asthma. We hypothesized that there would be no association between MSG intake and asthma. Using data from Jiangsu Nutrition Study (JIN), the current study aimed to test this hypothesis in the Chinese adult population, as well as to assess the relationship between dietary patterns and asthma.

## Materials and Methods

### Subjects

The JIN cohort study of persons aged 20 years or older and the methods of sampling have been described previously [23]. In 2002, 2849 adults aged 20 and above living in two cities and six rural areas took part in a Chinese National Nutrition and Health Survey. In 2007, an attempt to re-contact all original participants was made. Some had either moved to other cities for temporary work or moved to other streets within the urban area. Therefore, of the original 2849 participants, 1682 were identified for follow-up. Overall, 1492 (52.3% of the original cohort) participated in the follow-up interview, and of these 24 participants did not answer the question regarding to asthma. In total 652 men and 834 women (total n = 1486) had information on MSG intake and asthma. Compared with the retained participants, those lost to follow-up, were generally younger (45.5 vs 49.3 years) but there were no differences in the mean BMI or energy intake. The study was conducted according to the guidelines laid down in the Declaration of Helsinki and all procedures involving human subjects/patients were approved by Jiangsu Provincial Centre for Disease Control and Prevention. Written informed consent was obtained from each participant.

### Data Collection and Measurements

Participants were interviewed at their homes by health workers using a standard questionnaire in 2002 and 2007 [23]. The health workers were intensively trained in all aspects of data collection.

#### Outcome variable-Asthma at follow-up

In 2007, each participant was asked for information regarding their history of asthma. The respondents were asked if they had been diagnosed with asthma by a doctor. If the answer is “Yes”, the participants were further asked about the date of the diagnosis.

#### Dietary intake

In 2002, dietary intake patterns during the previous year were investigated by a series of detailed questions about the usual frequency and quantity of intake of 33 food groups and beverages. The food frequency questionnaire (FFQ) has been validated [24] and reported to be a useful method for the collection of individual food consumption information in face-to-face interviews, but not in self-administered surveys, due to the current educational level of the majority of the Chinese population.

#### Baseline MSG intake

To determine the amount of MSG and other seasonings consumed by individuals, each household was specifically asked about their usual monthly consumption of these items. Individual consumption of MSG was calculated according to the total amount of MSG consumed in the household divided by the number of individuals per household and then adjusted for the proportion of the household energy intake energy consumed by each individual. Average total glutamate intake was also calculated by adding the glutamate concentrations of all foods/seasonings consumed by an individual per day. Nutrient (e.g. sodium, potassium, fibre) and vegetable oil intakes were assessed using a 3-day weighed food diary which recorded all foods consumed by each individual, on three consecutive days; this was done to confirm the intakes reported from the FFQ data. We did not consider under- and over-report of energy intake to be an issue, because upon reviewing the food diaries with the subjects, the health workers would clarify any intake value for a particular food that fell below or above the usual value reportedly consumed by the population within the region. Not everyone was asked for dietary information at follow up (FFQ was only used among those 15 years and above) however the household MSG intake was determined, thus mean household MSG intake was calculated. Food consumption data were analyzed using the Chinese Food Composition Table. In the current analysis, energy and nutrient intake was calculated using a 3-day weighted food diary.

#### Dietary patterns

Dietary patterns were identified by factor analysis based on food intake measured by the food frequency questionnaire (FFQ), using standard principal component analysis as described elsewhere [25]. In short, four food patterns were obtained - Factor 1 (‘macho’) was characterized by various kinds of animal foods and alcohol, i.e. foods commonly consumed by men; Factor 2 (the ‘traditional’ pattern) loaded heavily on rice, fresh vegetables and inversely on wheat flour; Factor 3 (‘sweet tooth’) contained cake, milk, yoghurt and drinks; and Factor 4 (‘vegetable rich’ pattern) included whole grains, fruits, root vegetables, fresh and pickled vegetables, milk, eggs and fish. The four factors explained 28.5% of the variance in intake.

#### Other lifestyle factors

Cigarette smoking was assessed by asking the frequency of daily cigarette smoking. Information on passive smoking was asked. Eating out was assessed by asking whether individuals ate out on a frequent basis and was coded as yes or no. Alcohol consumption was assessed by asking the frequency and amount of alcohol consumed. Education was recoded into either ‘Low’ (illiteracy, primary school); ‘Medium’ (junior middle school); or, ‘High’ (high middle school or higher), based on six categories of education levels in the questionnaire. Occupation was recoded into ‘Manual’ or ‘Non-manual’ based on a question with 12 occupational categories. Information on household income was also asked and was categorised into ‘Low’; ‘Medium’; or “High’.

#### Anthropometric measurements

In both 2002 and 2007, anthropometric measurements were obtained using standard protocols and techniques. Body weight was measured in light indoor clothing without shoes to the nearest 100 grams. Height was measured without shoes to the nearest mm using a stadiometer. Overweight was defined as BMI ≥25 kg/m^2^.

### Statistics

MSG intake was recoded into tertiles. Chi square tests were used to compare the difference between categorical variables, and ANOVA was used to compare differences in continuous variables between groups. Rare events logistic regression proposed by Gary King and Langche Zeng [26] was used to determine the association between MSG intake (tertiles) and asthma adjusted for age, education, occupation, smoking, alcohol drinking, overweight, energy intake and food patterns. In assessing the association between food patterns and asthma, baseline food patterns scores were used as continuous variables in rare events logistic regression models. We tested for linear trends across categories of MSG intake by assigning each participant the median value for the category and modelling this value as a continuous variable instead of using ordinal numbers (1,2, 3 for tertiles of MSG intake) because the difference of the median MSG intake is not 1 between tertiles 2 and 1, or between tertile 3 and 2. We also used MSG intake as a continuous variable in the multivariable rare events logistic regression. These multivariate rare event logistic regression models accounted for household cluster using *cluster()* option in *relogit* syntax in order to have robust standard errors. All the analyses were performed using STATA 12 (Stata Corporation, College Station). Stata code relogit written by Gary King and Langche Zeng was used to perform rare events logistic regression. Statistical significance was considered to be when p<0.05 (two sided).

## Results

The mean intake of MSG for the entire population was 3.1 g/day (SD, 4.0). Of the 1486 participants, 108 reported no use of MSG and the median intakes across the tertiles were 0.9, 2.6, and 5.6 g/d, respectively. Table 1 shows the cross-sectional associations between MSG intake and socio-demographic and lifestyle factors. MSG intake was positively associated with smoking and alcohol drinking (p<0.001). No significant difference in energy was found across MSG intake tertiles. At baseline, the overall prevalence of overweight/obesity (BMI ≥25 kg/m^2^) was 29.7%.

**Table 1:** Sample characteristic according to MSG intake tertiles among Chinese adults (Jiangsu Nutrition Study) (n = 1486).

	T1(n = 496)	T2(n = 495)	T3(n = 495)	p
Age (years) (mean, SD)	48.7(13.4)	48.8(13.7)	48.9(13.2)	0.978
BMI (kg/m^2^ ) (mean, SD)	23.8(3.5)	23.6(3.5)	23.1(3.2)	0.002
MSG intake (g/d) (median, interquartile range)	0.9(1.0)	2.6(1.1)	5.6(3.7)	<0.001
Energy intake (kcal/d) (mean, SD)	2288(673)	2312(640)	2365(631)	0.162
Smoking (%)				
None	76.4	73.9	65.5	<0.001
<19	13.1	13.7	14.5	
>20	10.5	12.3	20.0	
Drinking (%)				
None	80	75.4	69.2	<0.001
1–2 per week	6.3	7.1	4.7	
3–4 per week	3.4	4.4	4.9	
Daily	10.3	13.1	21.3	
Education (%)				
Low	55.2	50.1	48.6	0.024
Medium	30	38.8	37.2	
High	14.7	11.1	14.2	
Income (%)				
Low	40.9	20.3	12.8	<0.001
Medium	33.2	36.8	32.5	
High	25.9	42.9	54.7	
Manual job (%)	53.7	50.3	45.7	0.039
Men (%)	35.1	43	53.5	<0.001
Overweight (BMI>25 kg/m^2^) (%)	31.7	29.9	27.5	0.362

Of the sample, 20 (1.4%) participants reported ever having asthma (1.2% in men, 1.4% in women). Two participants reported having been diagnosed with asthma during the follow up (after 2002). Across the tertiles of MSG intake, the number of asthma cases seemed to increase; however it is not statistically significant. The prevalence of asthma across the MSG tertiles was 1.0%, 1.2%, and 1.8% (p = 0.516) (Table 2). The mean age of those reported asthma was 62.6(SD 11.6) years. Only two participants aged below 50 years reported having asthma.

**Table 2:** Prevalence of asthma by tertiles of monosodium glutamate (MSG) intake tertiles among adults in China (Jiangsu Nutrition Study) (n = 1486).

	MSG intake tertiles				
	T1	T2	T3	Total	p
Number of participants	496	495	495	1,486	
Number of asthma cases	5	6	9	20	
Prevalence of asthma (%)	1.0	1.2	1.8	1.4	0.516

In multivariate rare events logistic regression analysis adjusting for age, gender, overweight/obesity, lifestyle factors as well as dietary factors including dietary patterns (Table 3), baseline MSG intake was not significantly associated with asthma. Across tertiles of MSG intake, odds ratio (OR) for asthma was: 1, 1.09(95%CI: 0.33–3.55), and 1.52(0.45–5.12) (p for trend 0.423). Using MSG intake as continuous variable, there was no significant association between MSG consumption and asthma (OR 1.04, 95% CI 0.99–1.09, p = 0.092). There was no association between MSG and asthma regardless of whether the analysis was based on continuous or tertiles of MSG intake.

**Table 3:** Odds ratio (95%CI) for asthma according to MSG intake tertiles among Chinese adults (Jiangsu Nutrition Study) (n = 1486).

	T1	T2			T3			*p* for trend
Model 1^a^	1.00	1.23	0.38–4.04		1.94	0.63–5.95		0.230
Model 2 b	1.00	1.19	0.36–3.94		1.93	0.58–6.40		0.218
Model 3 c	1.00	1.09	0.33–3.55		1.52	0.45–5.12		0.423

a Model 1 adjusted for age, gender and energy intake.

b Model 2 additional adjustment for smoking, alcohol drinking, income, manual job, overweight/obesity.

c Model 3 additional adjustment for dietary patterns but exclude alcohol drinking.

Table 4 shows that age was positively associated with asthma in the multivariate analysis. There was a significant positive association between ‘traditional’ food intake pattern and asthma. The ORs for asthma for the traditional food pattern score (1 SD change) was 2.25(1.45–3.51). No interaction between ‘traditional’ food pattern and age was found in relation to asthma (data not shown). The associations between ‘macho’, ‘sweet tooth’, and ‘vegetable rich’ food pattern and asthma were not significant. There was no association between gender, overweight, smoking, alcohol and asthma. The prevalence of asthma was 1.0% among smokers and 1.5% among non-smokers (p = 0.419). Because the number of asthma cases was small, we performed a parsimonious model including only age, MSG and ‘traditional’ food pattern. The associations between MSG, ‘traditional’ food pattern and asthma did not change.

**Table 4:** Lifestyle and sociodemographic factors associated with asthma in Chinese adults (Jiangsu Nutrition Study) (n = 1486).

	OR	95%CI
Age	**1.07**	**1.04–1.11**
Sex (men vs women)	0.93	0.35–2.46
Smoking		
None	1.00	
1–19 cigarettes/d	0.57	0.06–4.99
≥20 cigarettes/d	1.33	0.34–5.71
Income		
Low	1.00	
Medium	1.27	0.45–3.64
High	0.41	0.10–1.63
Manual job	0.89	0.34–2.37
BMI≥25 kg/m2	0.87	0.31–2.45
Food pattern scores		
Macho pattern	0.79	0.31–2.00
Traditional pattern	**2.25**	**1.45–3.51**
Sweet tooth pattern	1.26	0.76–2.07
Vegetable rich pattern	0.85	0.50–1.47

OR adjusted for energy and MSG intake in addition to all the variables in the table.

Because ‘traditional’ food pattern was positively associated with animal fat intake in the sample [27], we then further explored the association between animal fat intake and asthma. Comparing the second/third tertile with the first tertile of animal fat intake (percentage of total fat), the OR for asthma was 3.36 (95% CI 0.91–12.36) after adjusting for energy intake, age, gender, income and BMI. Adding animal fat intake into a parsimonious model including energy intake, age, gender, income and BMI, the OR for asthma of ‘traditional’ food pattern decreased 2.6% but remained significant (data not shown). Adjusting animal fat intake, the association between MSG intake and asthma remained non-significant. We further assessed the association between intake of rice, wheat flour and vegetable and asthma. Only vegetable intake was found to be significantly positively associated with asthma (OR 1.26, 95%CI 1.08–1.48 for 1SD (253g/d) increase of vegetable intake).

## Discussion

In this study, we found no association between the intake of MSG and asthma among Chinese adults. There was a significant positive association between ‘traditional’ food pattern, animal fat intake and asthma. No association between ‘macho’, ‘sweet tooth’, ‘vegetable rich’ food patterns and asthma was found. To our knowledge, this is the first large population study with detailed information on nutritional assessments to report on the relationship between MSG intake and asthma.

The prevalence of asthma in the Chinese population is lower than Western populations. The prevalence of asthma (1.4%) in our sample is consistent with that of other studies in China, although because of age related differences in the cohorts studied, and the increase in asthma with increasing age, direct comparison is not possible. In rural Beijing, 3.4% of women and 2.5% of men aged 50–59 years reported having had an asthma attack during the previous 12 months [4].

In daily life, it is not good enough to say a food is good or bad without mention the quantity consumed. In our sample, even the mean MSG intake is high compared with many developed countries, we did not observe a significant increased risk of asthma. The non-positive association between MSG intake and asthma in this study is consistent with several clinical trials [18]–[20] as well as an animal study [22]. Although some studies suggested a link between MSG intake and obesity [28], [29], we did not find such association in this cohort [23]. A recent study in Vietnam also found no association between MSG and overweight [30]. We have previously reported a positive association between MSG intake and increase in blood pressure [31]. A positive association between MSG and metabolic syndrome was reported in a highly selective rural Thai population [32].

Inconsistent with the other studies, smoking or overweight were not associated with asthma in the study. In western countries, findings from seven prospective epidemiologic studies suggest that overweight and obesity are positively associated with the risk of incident asthma [33]. Association between overweight and asthma is inconsistent in Asian populations. Overweight and obesity do not lead to asthma in Asian immigrant children in USA [34]. A high BMI increases the risk of having asthma in symptomatic adolescent females but not in adolescent males in Taiwan [35]. During the past decades, China has experienced substantial nutrition transition. Overweight/obesity and other chronic diseases have become major health problems, but appear not to be accompanied by an increase in asthma. There is some suggestion that obesity duration is related to pulmonary function impairment in obese subjects [36] and perhaps the obesity duration is not long enough in China to cause asthma.

China is one of the countries with high prevalence of smoking. It is reported that 28.1% of adults in China (52.9% of men and 2.4% of women) were current smokers [37]. In our sample, the proportion of smokers was very similar to the national figure. A low prevalence of asthma in China precludes the possibility of smoking as one of the major contributor to asthma in the population. It is thus not unexpected that in our study we found no association between smoking and asthma.

Like many other studies, we found a positive association between age and asthma in the study. A positive association between ‘traditional’ food pattern and asthma could not be explained by its correlation with age, as no interaction between ‘traditional’ food pattern and age was observed. Compared with western countries China has a low prevalence of asthma, one would expect a protective effect of traditional food pattern on asthma if diet plays an important role. We have previously reported the association between dietary patterns and weight change over five years. The mean weight gain in the sample over five years is less than 1 Kg. The ‘traditional’ food pattern is inversely associated with weight gain [27]. However, this association does not translate into a relationship with asthma. Interestingly in the sample ‘traditional’ food pattern was positively associated with animal fat intake. Consistent with current knowledge [38], animal fat intake was positively and plant fat inversely associated with asthma in our study. It could be that the positive association between ‘traditional’ pattern and asthma is due to its high intake of animal fat. However, adding animal fat intake in the multivariable model the OR for asthma of ‘traditional’ food pattern only decreased 2.6%. Dietary patterns are often confounded by socio-economic and lifestyle factors so their effect estimates on the health outcomes could be easily attenuated [39]. It could be that the ‘traditional’ food pattern is only a marker of other unmeasured factors, eg. pesticide exposure [40]. In the province, based on a very large population survey it was reported that the prevalence of asthma among children increased from 1.9% in 1990 to 2.4% in 2000 [41]. Although the causes of this increase maybe complex, urbanization and industrialization related exposure to air pollution may be part of the reason. The positive association between vegetable intake and asthma was unexpected. In the sample vegetable intake was positively related to income. As this sample comprises mainly participants from the rural area, a high income may mean factories related job and exposure to air pollution. It is also possible that pesticides or chemical residue in the vegetables may play a role in the link.

An antioxidant rich diet is protective against asthma [8]. In our study, we failed to find any association between vegetable rich food pattern and asthma. Several other studies have also shown no association between a high intake of fruit and vegetables and asthma [12]. Among Singapore Chinese, a dietary pattern characterized by meat and starchy food intake is related to respiratory symptoms [42]. Using alcohol to treat asthma is an ancient practice. The association between alcohol and asthma has been reviewed in depth [43]. Moderate alcohol consumption has been shown to increase insulin sensitivity [44]. Insulin resistance is associated with asthma among children and adults [45], [46]. In China, during the past three decades of rapid nutrition transition, the consumption of both alcohol and meat increased substantially. A sustained low prevalence of asthma suggests alcohol and meat dietary pattern may not have an important role in the development of asthma in China.

One of the limitations of the study is the small number of asthma cases, and the somewhat limited power of the study sample. The sample has only a power of 14.9% in detecting the significant difference of the prevalence of asthma between the extreme tertiles of MSG intake. There is a high probability of type II error. We were not able to assess the association between MSG intake, dietary patterns and incident asthma or the aggravation of asthma. However, we did find significant associations between dietary pattern and prevalent asthma in the sample. The second limitation is the measurement of individual MSG intake. It is based on household monthly MSG consumption. In large population study, accurate of MSG intake is very difficult. The third limitation of the study is the use of self-reported doctor diagnosed asthma. There is possibility of misclassification. However, the prevalence of asthma in the sample is similar with other studies in China. The low prevalence of asthma is unlikely due to lack of access to health service, as the region is one of the richest in China with a well established health service system. The fourth limitation is the high rate of drop out. However, those dropped out were in general young migrant workers. It is unlikely that this drop out was related to asthma. Furthermore, we cannot exclude the possibility that people with asthma may avoid monosodium glutamate after asthma has been diagnosed. Finally, as a study using factor analysis to determine the dietary patterns, the study has limitations which are common in many dietary pattern studies [47], for example, the dietary pattern solutions are strongly affected by subjective analytic but important decisions, such as the number of factors to extract, the variance explained. The strength of the study is the use of detailed information of nutritional intake based on 3-day weighted food records. Overweight was assessed by measured height and weight.

In conclusion, there was no significant positive association between MSG intake and asthma among Chinese adults. ‘Traditional’ food pattern was positively associated with asthma, which was partly explained by animal fat intake. No association between ‘vegetable rich’, ‘macho’, and ‘sweet tooth’ dietary patterns and asthma were found. Further prospective studies with large samples and younger age groups are needed.
